# Reproductive compensatory photosynthesis in a semi-arid rangeland bunchgrass

**DOI:** 10.1007/s00442-023-05341-w

**Published:** 2023-03-02

**Authors:** Erik P. Hamerlynck, Rory C. O’Connor, Stella M. Copeland

**Affiliations:** 1 USDA-ARS Research Ecologist, 67826A, OR-205, Burns, OR 97720 USA; 2 USDA-ARS Rangeland Scientist, Eastern Oregon Agricultural Research Center, 67826A, OR-205, Burns, OR 97720 USA

**Keywords:** Crested wheatgrass, Gas exchange, Herbivory, Reproductive effort, Sagebrush steppe

## Abstract

While increased foliar photosynthesis is well documented across many plant species in response to diverse modes of herbivory, the compensatory ability of photosynthetically active reproductive structures is unknown. To address this, we partially defoliated basal florets in seed heads of crested wheatgrass (*Agropyron cristatum* (L.) Gaertn.), an exotic Eurasian perennial bunchgrass widely distributed across North American sagebrush steppe. We followed direct and indirect responses by tracking post-clipping photosynthesis in clipped basal and unclipped distal florets, respectively, and comparing these to similar florets on unclipped seed heads. Compensatory photosynthesis was apparent 24 h after clipping; over the pre-anthesis period, clipped basal floret photosynthesis was + 62%, stomatal conductance was + 82%, and PSII photochemical yield was − 39% of unclipped controls. After anthesis, intact florets distal to clipped florets had modestly higher photosynthetic rates compared to controls, while basal floret rates did not differ between treatments. Compensatory photosynthesis reduced intrinsic water use efficiency (iWUE; photosynthesis/stomatal conductance) 68–40% below controls over pre- and post-anthesis periods, respectively. Specific mass (dry mass/area) of clipped florets was − 15% of controls, while florets distal to these had specific mass 11% greater than distal or basal florets on unclipped seed heads. These results suggest damaged basal florets provided carbon to unaffected distal florets. This could explain crested wheatgrass’s ability to produce viable seeds under conditions limiting to native bunchgrasses, and presents a novel mechanism germane to the development of convergent drought- and grazing-tolerance traits important to arid and semi-arid rangeland plant community resilience to climate variability.

**Supplementary Information:**

The online version contains supplementary material available at 10.1007/s00442-023-05341-w.

## Introduction

It has long been recognized that phytophagous insects are important herbivores in the arid and semi-arid rangelands of North America (Mack and Thompson 1982; Evans and Seastedt 1995). Some of these inconspicuous herbivores specifically target grass reproductive structures, giving them an outsized effect on plant reproductive success, population growth and subsequent plant–plant interactions that shape plant community dynamics (Otte and Joern 1976; Joern 1982; Trumble et al. 1993; Hanley et al. 1995; Joern and Mole 2005; LaPierre et al. 2015). There is evidence that drought and grazing impart plant trait characteristics that co-enhance tolerance to both stresses, and that grazing selects genotypes that respond better to drought by enhancing sexual reproduction (Adler et al. 2004; Quiroga et al. 2010). However, no studies have assessed the direct effects of herbivory of bunchgrass reproductive structures. While a diverse array of poly- and oligophagous insects, especially adult beetles and lepidopteran larvae, have been documented to graze florets and developing seeds in sagebrush steppe grasses (Youti et al. 1987), the importance of this activity to plant reproductive effort and any associated reproductive photosynthetic responses has not been documented in rangeland bunchgrasses.

It is well established that many arid and semi-arid perennial bunchgrasses increase leaf-level net photosynthetic assimilation rates (*A*_net_) for several days following defoliation (Caldwell et al. 1981; Detling and Painter 1983; Nowak and Caldwell 1984; Wallace et al. 1984; Senock et al. 1991; Doescher et al. 1997; Zhao et al. 2008; Hamerlynck et al. 2016a). While this transient assimilatory increase may contribute to grazing and drought tolerance in seedlings (Hamerlynck et al. 2016a; Denton et al. 2018), it does not seem to do so in established adult plants, where longer-term post-defoliation adjustments in biomass allocation modulates overall herbivory tolerance (Caldwell et al. 1981; McNaughton 1983; McNaughton et al. 1983; Nowak and Caldwell 1984; Richards 1984; Hamerlynck et al. 2016b). As such, Hamerlynck et al. (2016a) conjectured that compensatory photosynthesis is a legacy response held over from seedling to adult demographic phases, and suggested it might play a role in some other aspect of adult plant function. If insect floral grazing is important to dryland bunchgrass functional ecology, compensatory photosynthesis in reproductive structures could be an adaptive response to such herbivory. Rangeland bunchgrass population dynamics overwhelmingly rely on sexual reproduction and establishment from seed (Smith et al. 1997; Liston et al. 2003; Hamerlynck and Davies 2019). Photosynthetic activity by the seed head itself significantly contributes to seed energetic provisioning and reproductive effort, indeed often exceeding flag leaf contributions (Hamerlynck et al. 2019; Hamerlynck and Ziegenhagen 2020; Hamerlynck and O’Connor 2021, 2022). Moreover, there is evidence in other graminoid species that there is considerable competition within the seed head for maternal carbon. This is evidenced by decreasing seed mass and seedling success going from basal to distal positions in the seed head (McDonald et al. 1996), and by increased mass during grain filling in the seeds that remain following removal of neighboring seeds (Belsky 1986; Warringa et al. 1998). These latter findings suggest that, in addition to immediate repair following damage, compensatory reproductive photosynthesis could contribute to improved seed quality that could offset the reduction in overall reproductive propagules following floret grazing.

Here, we present results of a study assessing the photosynthetic compensatory responses to partial floret and flag leaf removal in the perennial bunchgrass, crested wheatgrass (*Andropogon cristatum* (L.) Gaetern.), a widely distributed Eurasian exotic and important restoration species in North American sagebrush steppe rangeland ecosystems (Hamerlynck and Boyd 2021). Previous work has shown crested wheatgrass seed heads on culms produced following whole plant clipping did not differ in photosynthetic performance compared to those from unclipped controls (Hamerlynck and Ziegenhagen 2020); however, this study did not address direct photosynthetic responses to loss of reproductive tissue. We experimentally clipped basal florets from seed heads and flag leaves on flowering culms in a full factorial design, and compared the photosynthetic activity of these and of florets located distally to these to distal and basal florets in seed heads on unmanipulated control culms. This allowed us to ascertain direct and indirect compensatory photosynthetic responses to floret tissue loss, the former via comparing basal florets, the latter via comparing distal florets. Given the high investment to seed head photosynthetic capacity (Hamerlynck et al. 2019) and significant reliance on seed head photosynthesis for seed provisioning and reproductive effort (Hamerlynck and O’Connor 2021, 2022), we specifically hypothesized that crested wheatgrass would increase photosynthetic rates after significant removal of seed head tissue. In addition, given the relatively minor role flag leaves play in crested wheatgrass reproductive effort (Hamerlynck and O’Connor 2021), we did not expect flag leaf removal to induce any compensatory response in seed head photosynthesis. To establish the importance of any reproductive compensatory photosynthesis in response to reproductive tissue loss, we compared the total floret area and aggregate floret specific mass (g m^−2^) of florets measured for photosynthetic gas exchange at the end of the reproductive growing season. If our clipping induced compensatory photosynthesis in either distal or basal florets in our seed heads, but did not increase total floret area or alter specific mass in similar florets in control plants, we would know it provided little benefit to overall reproductive allocation.

## Materials and methods

The study took place from 07-June-2022 to 15-July-2022 on the USDA Agricultural Research Center Northern Great Basin Experimental Range (NGBER; 119°43′W, 43°29′N), located about 70 km west of Burns, OR at 1402 m ASL. The site has a mean annual temperature of 14.8 °C, with daily average maximums of 28.7 °C in July to − 7.1 °C in January. Mean annual precipitation (MAP) is 278.4 mm, with 71% of this as rainfall distributed evenly across the November to May cool season period, with occasional snowfall over the coldest months. We sampled from the same crested wheatgrass (*Agropyron cristatum* (L.) Gaertn.) stand sampled by Hamerlynck et al. (2019), located in a large level area of intact sagebrush steppe, enclosed and protected from livestock grazing following completion of construction of five rainout shelters in 1994 (Svejcar et al. 1999). The grasses sampled established from existing local seed sources, and as such we could not ascertain what cultivar of crested wheatgrass is present in the study area. Total plant cover at the site is 31.1%, with 29.5% perennial plant cover, the bulk of which are perennial grasses (15.0%) and shrubs (9.6%, primarily Wyoming big sagebrush, *Artemesia tridentata* subsp. *wyomingensis*); the exotic annual grass, *Bromus tectorum*, is infrequent and sparsely distributed (0.01% cover; Hamerlynck et al. 2016b).

We obtained daily precipitation data from a National Atmospheric Deposition Program monitoring site (NADP site OR07; Burns Sagebrush) located ca. 100 m from the sampled stand of crested wheatgrass. To provide a soil moisture context in response to any observed precipitation, we randomly selected data from 22 volumetric soil moisture (*θ*; mol^3^ mol^−3^) and soil temperature (*T*_soil_) probes from an array of 60 at a soil moisture monitoring site located ca. 200 m from our sampling plot. Since October 2018, we have measured *θ* and *T*_soil_ within rooting zone soils at 10 cm depth under three different Great Basin bunchgrasses including crested wheatgrass using ECH_2_O 5TM probes, with data recorded every 4 h on Em50 dataloggers (Decagon Devices, Pullman, WA). We averaged each 4 h observation across all 22 probes, then averaged these over each 24 h period to estimate daily average *θ* and *T*_soil_. While this array does not monitor the actual plants sampled, the soils at this location are the same as the sampled plants are growing (for soil and probe installation details, see Hamerlynck and Ziegenhagen 2020 and references therein), and thus provides a sufficient information for soil moisture and temperature dynamics and associated plant responses to any observed precipitation events over the course of the study.

Ten individual crested wheatgrass plants were selected at random, with four flowering culms selected from the south-facing half of the bunchgrass. Each culm was marked with a numbered tag attached to the culm below the first true leaf down stem from the flag leaf. Prior to any clipping, the length and width of each seed head was measured to the nearest 0.1 cm. On June 14, 2022, the culms were randomly treated in four-way factorial clipping treatment: unclipped seed head and flag leaf (control), clipped seed head/unclipped flag leaf (clip/con), unclipped seed head/clipped flag leaf (con/clip), and clipped seed head and flag leaf (clip/clip). For clipped seed heads, we removed ca. 30–50% of all florets ca. 3 cm below the terminal distal portion of the seed head. Flag leaves were fully excised at the collar clasping the culm.

Photosynthetic gas exchange and chlorophyll fluorescence measurements were made using a LiCOR 6800 portable photosynthesis system (LiCOR Instruments, Lincoln, NE, USA). Photosynthetic measurements were made from 0830 to 1230 MDT, with a random sampling order to avoid any confounding diurnal variation in photosynthetic performance with any treatment effects. Prior to enclosure, the width of seed heads was measured to nearest 0.5 mm with a ruler to determine surface area and multiplied by the cuvette length (3 cm) to area correct gas exchange measurements. Saturating incident photosynthetic photon flux density (PPFD) of 1500 μmol m^−2^ s^−1^ was provided by a red/blue LED light source attached to cuvette, set to a default red:blue ratio of 9:1. Cuvette relative humidity was maintained at 40%, attained by mixing air between a desiccant column filled with Drierite with that passing through Nafion™ tubing immersed in a column of Nanopure deionized water. The temperature of a Peltier-exchange temperature control block was set to 25.0 °C, resulting in leaf temperatures of 24.0–30.0 °C as measured with a fine-wire thermocouple held against the underside of the sample, and leaf-to-air vapor pressure deficits of 1.1–3.0 kPa, depending on external conditions and the time of day. To minimize leak effects, a high-speed fan was set to maintain a pressure difference of 0.1 kPa between cuvette and the outside atmosphere. Reference cell CO_2_ concentration was set to 400 PPM, and reference and sample cells allowed to stabilize ([CO_2_] and [H_2_O] slope vs time less than 1.0 μmol min^−1^, with a standard deviation less than 0.2, then matched to a common air stream to eliminate reference and sample cell infrared gas analyzer (IRGA) differences. These protocols allowed the enclosed tissue to acclimate for ca. 120 s to cuvette conditions prior to measurement of net photosynthesis (*A*_net_; μmol m^−2^ s^−1^) and stomatal conductance to water vapor (*g*_s_; mol m^−2^ s^−1^). Immediately after photosynthetic gas exchange data was logged, light-adapted PSII photochemical yield (*ϕ*_PSII_) was determined by measuring chlorophyll fluorescence (*F*) with a LiCOR multi-phase fluorimeter integrated with the cuvette. A modulating beam of 5.0 μmol m^−2^ s^−1^ intensity modulated at 50 kHz was applied for 5 s to determine steady-state fluorescence yield (*F*_s_) under the incident PPFD of 1500 μmol m^−2^ s^−1^. This was followed by exposure to three successive flashes of a saturating actinic beam of 10,000 μmol m^−2^ s^−1^, each of 300 ms pulse width and a 90% red/10% blue light balance, modulated at 250 kHz with data gathered at 100 Hz to determine maximum light-adapted fluorescence yield (*F*_m_′), with *ϕ*_PSII_ calculated as *ϕ*_PSII_ = (*F*_m_′–*F*_s_)/*F*_m_′.

Photosynthesis measurements commenced June 15, 1 day after basal clipping, then repeated at 2, 3, 7, 10, 14, 17, 21, and 24 days post-clipping. Basal clipping occurred in the pre-anthesis stage, prior to grain filling (Hamerlynck et al. 2019); phenological stage was noted at each sampling date, with anthesis observed on June 28, 14 d after clipping with seed filling occurring from 17- to 24-d post-clipping. Gas exchange and chlorophyll fluorescence measurements were made at two locations on each seed head of the four treated culms; the first on the distal, top portion of the seed head, followed immediately by a second on the basal portion. On the last day of sampling, the terminal and basal florets of all plants were harvested, bagged, placed in a cooler, and subsequently scanned on a flat-bed scanner and measured for total silhouette area using WinRhizo image analysis software (Regent Instruments, Quebec, QE, Canada), then dried at 48 °C for at least 72 h, weighed to nearest 0.0001 g to determine aggregate floret specific mass (g m^−2^).

We used a split-plot repeated-measures analysis of variance (RM-ANOVA, Statistix v.8.0, Analytical Software, Tallahassee, FL, USA) to test for differences in distal and basal floret *A*_net_, *g*_s_, and *ϕ*_PSII_. As we were only interested in differences in direct and indirect compensatory photosynthetic responses compared to untreated controls in the same position on the seed head, we did not statistically compare distal and basal floret photosynthetic performance within- or between-treatment combinations. Following Hamerlynck and O’Connor (2022), we separately averaged distal and basal floret gas exchange and fluorescence measurements for each culm on each replicate plant across the pre- (1, 2, 3, 7, and 10 days after clipping) and post-anthesis periods (days 17, 21, and 24). This gave us three within-treatment phenological stages—pre-anthesis, anthesis, and post-anthesis—with equal sample sizes, avoiding an unbalanced RM-ANOVA design. Whole-plot between-treatment effects were basal floret clipping, flag leaf removal, and the clipping-by-removal interaction, using the clipping-by-removal-by-replicate plant interaction as the whole-plot error term. The sub-plot effects were phenological stage (pre-anthesis, anthesis, post-anthesis), and all two-way and three-way interactions with seed head clipping and flag leaf removal treatments, using the clipping-by-removal-by-stage-by-replicate plant interaction as the sub-plot *F* test error term. Of specific interest were the phenological stage-by-basal clipping and stage-by-flag leaf removal interactions, as these would show treatment-specific differences in basal and distal floret photosynthetic performance over the three stages in response to clipping. RM-ANOVA results were considered significant at an associated *p* value < 0.05; post hoc means testing was made using LSD, with α-adjusted to the appropriate attained *F* test *p* value.

As in Hamerlynck et al. (2016a), we used linear regressions of *A*_net_ to *g*_s_ to determine intrinsic water use efficiency (iWUE) of distal and basal florets integrated across the pre- and post-anthesis periods, using *F* test slope comparisons to test for differences in integrated iWUE between clipped and unclipped seed heads for each phenological stage (Statistix v8.0).

Harvested distal and basal total floret area and aggregate floret specific masses were analyzed using a split-plot repeated-measures three-way ANOVA (Statistix v8.0). In this case, we did statistically compare basal and distal total floret area and aggregate specific masses both within and between seed head clipping treatments. Whole-plot within-treatment effects were basal clipping, flag leaf removal, and their interaction effect, using the clipping-by-removal-by-replicate plant interaction effect as the *F* test error term. Sub-plot, repeated-measures effects were floret location (basal vs. distal), and the location-by-clipping, location-by-flag leaf removal, and the location-by-clipping-by-flag leaf removal interaction effects, using the location-by-clipping-by-flag leaf removal-by-replicate plant interaction effect as the sub-plot *F* test error term. ANOVA *F* test results were considered significant at an associated *p* value ≤ 0.05; post hoc means testing was made using LSD, with α-adjusted to the appropriate *F* test *p* value.

## Results

Flag leaf removal did not significantly affect any aspect of distal or basal floret photosynthesis (Table 1); as such, our subsequent presentation of results will focus only on the photosynthetic responses of distal and basal florets to clipping florets in the basal portion of the seed head.

**Table 1:** Repeated-measures ANOVA *F* test results comparing the effects of seed head basal clipping (clip) and flag leaf removal (-leaf) on distal (top) and basal (base) floret net photosynthesis (*A*_net_), stomatal conductance to water vapor (*g*_s_), and light-adapted PSII photochemical yield (*ϕ*_PSII_) between pre-anthesis, anthesis, and post-anthesis periods (stage)

Effect	Top *A*_net_	Top *g*_s_	Top *ϕ*_PSII_	Base *A*_net_	Base *g*_s_	Base *ϕ*_PSII_
Base clip (clip)	0.04_(1,35)_	0.01_(1,35)_	0.10_(1,35)_	2.09_(1,36)_	2.98_(1,36)_	**66.61**_**(1,36)**_
Remove leaf (-leaf)	0.01_(1,35)_	0.05_(1,35)_	0.19_(1,35)_	0.79_(1,36)_	0.72_(1,36)_	0.12_(1,36)_
Clip x -leaf	0.12_(1,35)_	0.33_(1,35)_	0.34_(1,35)_	0.01_(1,36)_	0.38_(1,36)_	1.61_(1,36)_
Stage	**8.02**_**(2,69)**_	**20.06**_**(2,69)**_	**58.52**_**(2,69)**_	**45.27**_**(2,71)**_	**14.07**_**(2,71)**_	**14.58**_**(2,71)**_
Stage x clip	2.42_(2,69)_	0.66_(2,69)_	**3.53**_**(2,69)**_	**4.45**_**(2,71)**_	**37.39**_**(2,71)**_	**49.31**_**(2,71)**_
Stage x -leaf	0.06_(2,69)_	0.55_(2,69)_	1.90_(2,69)_	0.01_(2,71)_	0.14_(2,71)_	0.24_(2,71)_
Stage x clip x -leaf	0.22_(2,69)_	0.12_(2,69)_	2.08_(2,69)_	0.34_(2,71)_	0.28_(2,71)_	0.35_(2,71)_

Bold *F* test results are significant at *p* ≤ 0.05; degrees of freedom presented parenthetically after each *F* test

**Fig. 1:**
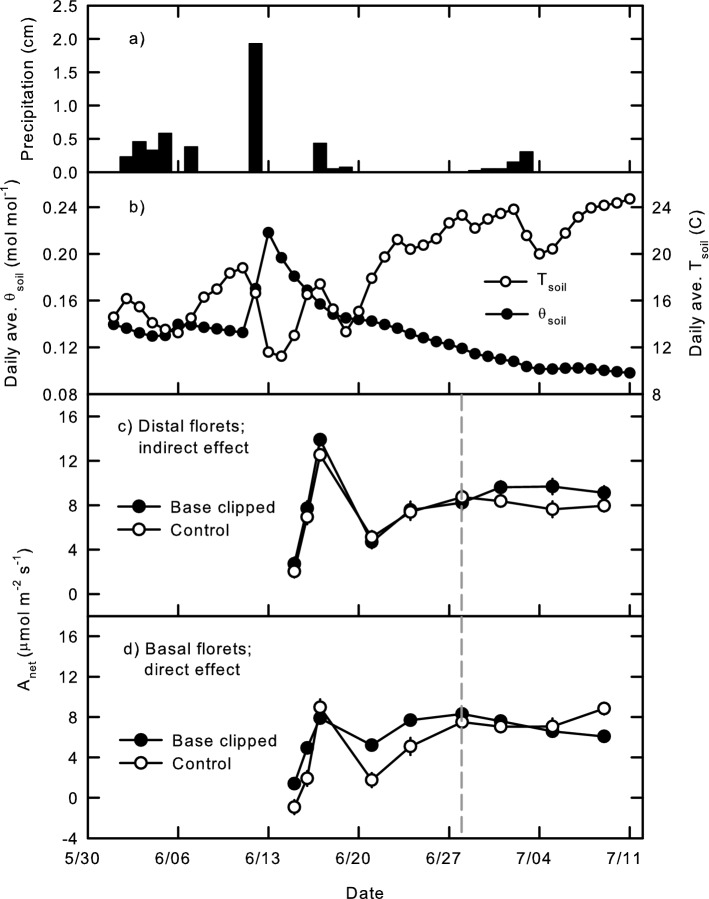
Daily (**a**) precipitation, average (**b**) rooting zone soil temperature (*T*_soil_) and volumetric soil moisture (*θ*_soil_), and average net photosynthetic assimilation rate (*A*_net_) of florets from the (**c**) top, distal, and (**d**) bottom basal portions of crested wheatgrass seed heads with clipped basal sections (closed symbols) and unclipped controls (open symbols). Each *T*_soil_ and *θ*_soil_ symbol is the average of 22 sensors, error bars indicate ± one S.E. of the mean. Basal florets from clipped seed heads are directly affected by tissue loss, distal florets on clipped seed heads are indirectly affected via basal clipping. Dashed gray line indicates occurrence of anthesis; pre-anthesis period to the left, post-anthesis seed filling to the right. Each *A*_net_ point is the mean of nine to ten measurements, four culms per plant

Several days of rain early in the reproductive period did not dramatically affect *θ*_soil_; however, a very large precipitation event (1.93 cm) on June 12 nearly doubled *θ*_soil_, and was followed by several days of decreased *T*_soil_ (Fig. 1a, b). The period of subsequent warming *T*_soil_ coincided with the first 3 days of post-clipping photosynthetic measurements, which showed concurrent increases in *A*_net_ in both distal (Fig. 1c) and basal florets (Fig. 1d). A series of small rain events from June 17 to 19 again reduced *T*_soil_ with only moderate effect on *θ*_soil_ (Fig. 1a, b), again with concurrent reductions in *A*_net_ and *g*_s_ (Fig. 1c, d), which then recovered to higher levels over the rest of the pre-anthesis period as *T*_soil_ warmed and *θ*_soil_ declined (Fig. 1). Anthesis was observed on June 28 (14 days after clipping; dashed line Fig. 1c, d), after which *T*_soil_ had warmed to a point that even fairly marked declines in *T*_soil_ following a few rains that did not affect the steady lowering of *θ*_soil_ (Fig. 1a, b) did not have any associated reductions in *A*_net_ over the post-anthesis period (Fig. 1c, d).

**Fig. 2:**
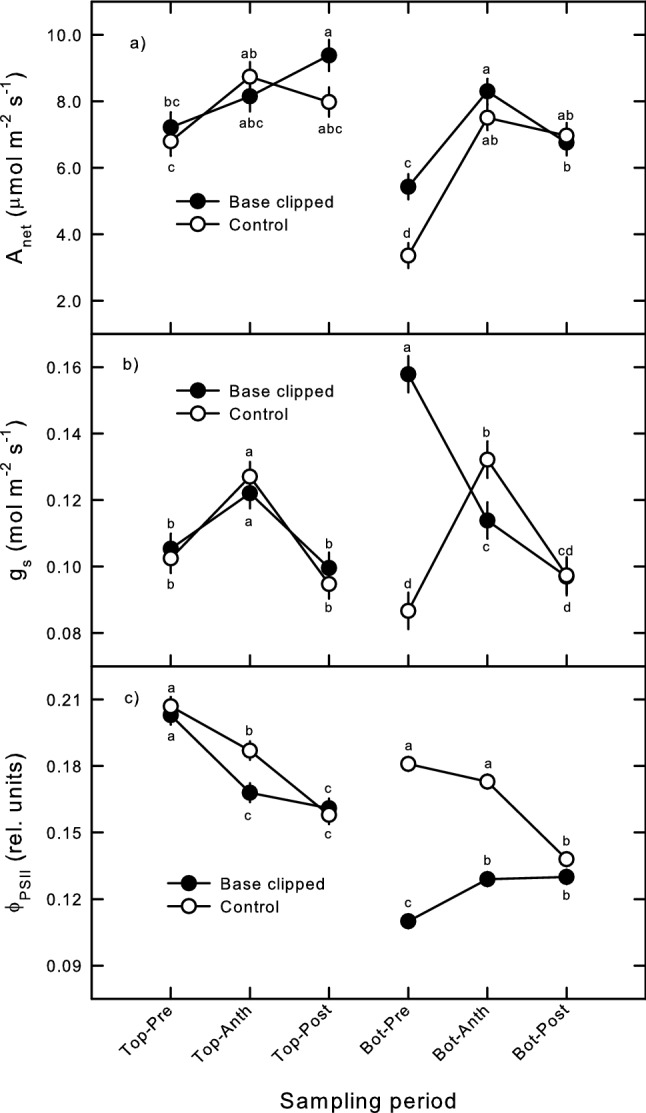
Pre-anthesis, anthesis and post-anthesis crested wheatgrass distal (top) and basal (base) floret **a** net photosynthesis (*A*_net_), **b** stomatal conductance to water vapor (*g*_s_), and **c** light-adapted PSII photochemical yield (*ϕ*_PSII_) on seed heads with clipped (solid symbols) and unclipped (open symbols) basal florets. Symbols are ANOVA means, error bars are ± 1 S.E.; letters differ significantly (LSD; *p* < 0.05)

Pooled across the study, both distal and basal floret *A*_net_ did not differ between clipping treatments, while basal floret *A*_net_ showed a significant phenological stage-by-clipping interaction effect (Table 1). In both positions, *A*_net_ increased from pre-anthesis lows to similar higher levels over the anthesis and post-anthesis periods (Fig. 2a). Over the pre-anthesis period, basal floret *A*_net_ was 62% higher in clipped florets compared to unclipped controls, but not at anthesis or over post-anthesis seed filling, when *A*_net_ was similar between treatments (Fig. 2a). In contrast, distal floret *A*_net_ did not differ between clipped and unclipped controls until the post-anthesis period, when *A*_net_ of distal florets on clipped seed heads were 19% higher than those on unclipped controls (Fig. 2a); however, this elevated distal floret *A*_net_ on clipped seed heads was only moderately significant (*p* = 0.098). Stomatal conductance to water vapor (*g*_s_) in distal florets was not affected by basal floret clipping, and only responded significantly to environmental variation across the three phenological stages (Table 1; Fig. 2b). Pre-anthesis *g*_s_ of clipped basal florets was significantly higher (+ 82%) than g_s_ in unclipped basal florets (Table 1; Fig. 2b), but did not differ at anthesis or across post-anthesis seed filling periods (Fig. 2b), driving a significant stage-by-clipping interaction effect (Table 1). Distal floret *ϕ*_PSII_ showed a significant stage-by-clip interaction (Table 1); this followed significant differences in control floret *ϕ*_PSII_ between pre-anthesis, anthesis, and post-anthesis periods, while *ϕ*_PSII_ in distal florets above clipped basal florets did not differ between anthesis and post-anthesis periods (Fig. 2c). Clipping basal florets induced a 39% decrease in *ϕ*_PSII_ compared to control floret *ϕ*_PSII_ over the pre-anthesis period, which narrowed to 26% lower by anthesis, then reaching unclipped control floret *ϕ*_PSII_ levels over the post-anthesis period (Fig. 2c). The large pre-anthesis reductions in *ϕ*_PSII_ drove both significant clipping treatment differences pooled across study and a stage-by clipping interaction (Table 1). The latter interaction was likely due to clipped basal floret *ϕ*_PSII_ not markedly changing from anthesis to post-anthesis, while unclipped control floret *ϕ*_PSII_ significantly declined 20% between these periods (Fig. 2c).

**Fig. 3:**
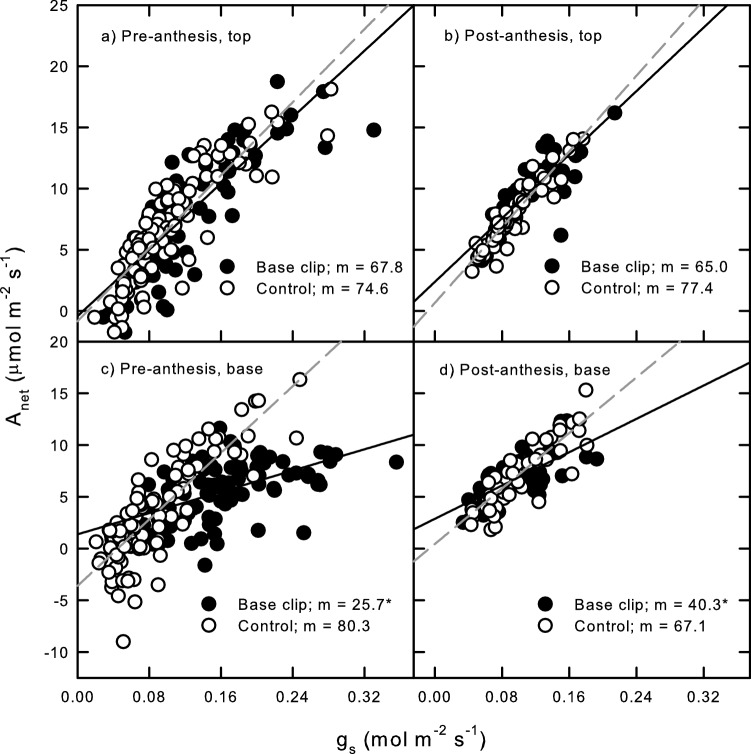
Linear regression of net photosynthesis (*A*_net_) with stomatal conductance to water vapor (*g*_s_) to determine intrinsic water use efficiency (iWUE) for pre- and post-anthesis (**a** and **b**) top, distal florets, and (**c** and **d**) pre- and post-anthesis basal florets of crested wheatgrass; slope values (m) following legend captions provide iWUE integrated across pre- and post-anthesis periods. *Indicates significant slope differences between base clipped and unclipped control seed heads (*p* ≤ 0.05)

Distal florets on basal-clipped seed heads had slightly lower iWUE compared to those distal to unclipped controls; however, these reductions did not significantly differ, across either the pre-anthesis (− 9%; *F*_1,191_ = 1.16; *p* = 0.282; Fig. 3a) or post-anthesis periods (− 16% *F*_1,110_ = 3.24; *p* = 0.075; Fig. 3b). In contrast, the markedly higher *A*_net_ and *g*_s_ in pre-anthesis clipped basal florets resulted in significantly lower iWUE than in unclipped pre-anthesis counterparts (− 68%; *F*_1,196_ = 63.32; *p* < 0.0001; Fig. 3c). Even though *A*_net_ and *g*_s_ were similar between clipped and unclipped basal florets over the post-anthesis period (Fig. 1b), iWUE was still 40% lower for clipped florets compared to controls (*F*_1,112_ = 12.96; *p* = 0.005; Fig. 3d). Though iWUE in clipped and control basal florets increased from pre- to post-anthesis periods, the slopes did not differ significantly between these periods for either treatment (*F*_1,153_ = 2.73; *p* = 0.1004 for clipped and *F*_1,155_ = 1.90; *p* = 0.17 for controls, respectively; Fig. 3c, d). Distal floret iWUE also did not differ between pre- and post-anthesis periods in clipped (*F*_1,145_ = 0.08; *p* = 0.781) or unclipped controls (*F*_1,156_ = 0.12; *p* = 0.735; Fig. 3a, b).

**Fig. 4:**
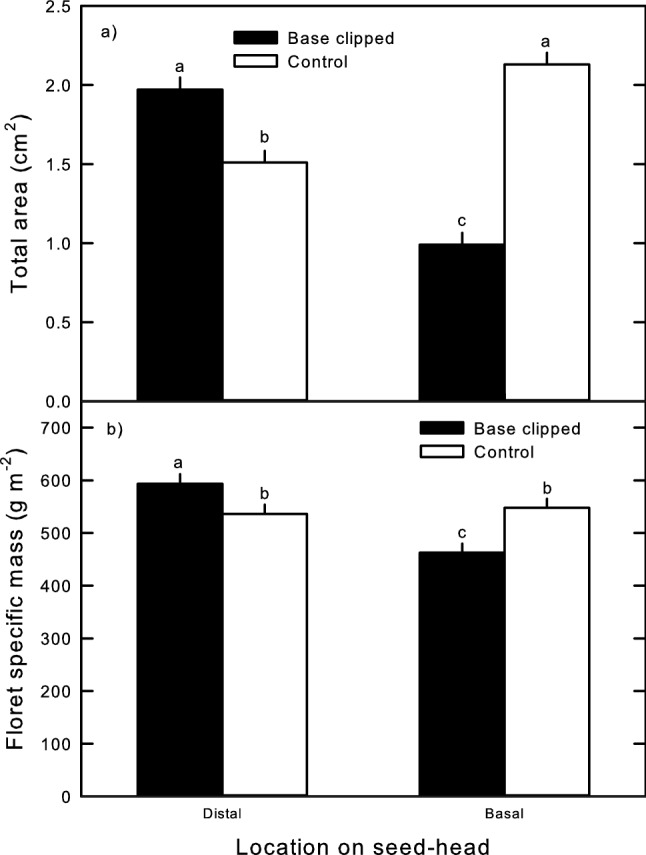
Repeated-measures ANOVA means of distal and basal **a** total floret area and **b** aggregate floret specific masses from crested wheatgrass seed heads with clipped (closed bars) and unclipped basal portions (open bars). Error bars are ± 1 S.E. Letters indicate significant differences in post hoc means tests results (LSD; *p* ≤ 0.05)

As with our gas exchange results, flag leaf removal did not significantly affect total basal or distal seed head area, or aggregate floret specific masses; however, there was a significant location and seed head location-by-clipping interaction effect in both (Table 2). In addition to our clipping treatment reducing final total basal floret area 52% compared to unclipped basal controls (Fig. 4a), the total area of distal florets on clipped seed heads was 30.3% greater than those distal to unclipped basal florets, and were similar to areas of unclipped basal florets (Fig. 4a). Greater total distal floret areas on clipped seed heads were concurrent with significantly greater aggregate floret specific masses, which were 11% greater than distal florets on unclipped seed heads (Fig. 4b); clipped basal floret specific masses were 14.6% lower than their unclipped counterparts (Fig. 4b). In addition, specific masses of distal and basal florets on unclipped seed heads did not differ, and were significantly lower than specific masses of intact distal florets from clipped seed heads (Fig. 4b). Thus, only the distinct positional differences in floret specific mass in clipped seed heads underlay the significant location effect (Table 2; Fig. 4b).

**Table 2:** Repeated-measures ANOVA *F* test results comparing total floret area and aggregate floret specific mass responses to seed head basal clipping (Clip; clipped vs. control), flag leaf removal (Leaf; removed vs. control) and floret location (Loc; distal vs. basal); bold results are significant at *p* ≤ 0.05, degrees freedom presented parenthetically after each effect

Effect	Area	Specific mass
Clip_(1,35)_	2.13	0.10
Leaf_(1,35)_	0.01	0.01
Clip x leaf_(1,35)_	0.15	0.07
Loc_(1,34)_	**5.84**	**11.24**
Loc x clip_(1,34)_	**112.22**	**15.93**
Loc x leaf_(1,34)_	0.80	0.67
Loc x clip x Leaf_(1,34)_	0.83	0.92

## Discussion

As hypothesized, clipping florets induced compensatory increased photosynthetic rates compared to unclipped controls, but was expressed at different locations and degrees over different phenological stages. Photosynthetic compensation was most strongly expressed over the pre-anthesis period in basal florets directly affected by clipping, with some evidence of modest indirect compensatory photosynthesis over the post-anthesis in unclipped florets distal to clipped basal florets (Fig. 1, 2). In contrast to previously documented instances of compensatory photosynthesis in perennial grasses, which typically only become evident several days to a week after tissue loss (Caldwell et al. 1981; Detling and Painter 1983; Nowak and Caldwell 1984; Senock et al. 1991; Zhao et al. 2008; Hamerlynck et al. 2016a), crested wheatgrass basal florets increased *A*_net_ and *g*_s_, with a marked decline in *ϕ*_PSII_ within one day following clipping (Figs. 1, 2). These results stand in contrast to several foliar studies, some of which found little change in *g*_s_, and an increase in photosynthetic enzyme activity and associated increases in *ϕ*_PSII_ and photosynthetic capacity (Thompson et al. 2002; Retuerto et al. 2004; Moustaka et al. 2021), and others that showed declines in *ϕ*_PSII_ were concurrent with decreased photosynthetic capacity following defoliation (Nabity et al. 2009; Huang et al. 2013; Liu et al. 2019). Higher *g*_s_, especially over the first day following removal (data not shown), was no doubt influenced by evaporative losses accompanying tissue removal. However, we observed well-developed callus formation of clipped florets 3 days following clipping (Hamerlynck, personal obs.), and thus for at least three of the five pre-anthesis sampling days, we are sure *g*_s_ in clipped were truly higher than in unclipped basal florets. Loss of *ϕ*_PSII_ yield was the most marked response following clipping, and, unlike photosynthetic gas exchange, was decidedly not compensatory. Such reductions in *ϕ*_PSII_ have been noted as a direct response to both internal and external insect leaf herbivory (Nabity et al. 2009; Huang et al. 2013; Liu et al. 2019), and may thus represent a general wounding response. It may also be that reduced *ϕ*_PSII_ could reflect photochemical adjustment to the altered physical structure of the seed head. In ears of wheat, which are remarkably similar to crested wheatgrass seed heads, there is considerable self-shading, and changing wheat ear display is known to influence light penetration and enhance photosynthetic activity in these structures (Wechsung et al. 2001). Possibly the removal of florets and parts of florets increased light penetration to the remaining tissue, inducing photoprotective mechanisms (Demming-Adams and Adams 1992; Osmond 1994). Indeed, declining *ϕ*_PSII_ in both control basal florets, as well as distal florets observed going into the post-anthesis period (Fig. 2) may reflect not only declining soil water availability (Fig. 1), but increased light penetration into the seed head as the seed head display opens in response to anthesis and fertilization. This agrees with assertions by Hamerlynck et al. (2019), who suggested such changes in seed head structure may also affect the balance between diffusive and enzymatic limitations to photosynthetic activity in this grass.

Unlike seedling studies which showed clipping increased leaf-level iWUE (Hamerlynck et al. 2016a), reproductive compensatory photosynthesis dramatically decreased iWUE compared to unclipped controls over both pre- and post-anthesis periods (Fig. 3). This suggests crested wheatgrass minimized diffusive limitations to carbon uptake following floret clipping, and that compensatory photosynthesis does not have consistent consequences to basic carbon/water trade-off across demographic stages, or possibly within the plant body itself. Unclipped floret iWUE is also consistent with spot measurements of whole seed head iWUE observed in Hamerlynck et al. (2019), which were on par with its own flag leaves and generally higher than iWUE attained by native bunchgrasses. This suggests that while compensatory photosynthetic upregulation in crested wheatgrass may reduce reproductive iWUE, it did to levels typically attained by native grass seed heads (Hamerlynck et al. 2019). Currently, we do not know if native grasses display reproductive compensatory photosynthetic upregulation. If tissue loss proportionally reduces iWUE in native grass florets to the degree apparent here in crested wheatgrass, it may be that effective compensatory responses in native grasses may be limited to years of high rainfall, and that reproductive herbivory in dry conditions could have a more marked effect on native grass reproductive effort than in crested wheatgrass, which is capable of consistently producing viable seed cohorts that can establish and persist through drought conditions (Hamerlynck and Davies 2019).

The marked increase in total area and aggregate specific mass of florets distal to clipped basal florets and the marked reduction in clipped basal floret specific mass strongly suggest a compensatory reproductive response to reproductive tissue loss. As alluded to above, the higher pre-anthesis *A*_net_ in clipped basal florets was a likely a direct repair response to tissue loss, similar to that observed in foliar studies (Briske and Richards 1995). However, this strong compensatory *A*_net_ response was followed by loss of floret specific masses compared to unclipped basal florets (Fig. 4). This suggests some of the carbon assimilated by damaged basal florets may have been utilized to enhance growth of the undamaged distal florets. In rangeland grasses, the number of florets is determined by prevailing soil moisture and temperature conditions over the “boot stage”, when the seed head is still enclosed by the portion of the flag leaf clasping the main culm (McDonald et al. 1996). As we measured only fully emerged seed heads, it is unlikely additional distal florets were produced following clipping. Rather, it may be clipped culm distal florets grew to a larger size over the pre-anthesis period compared to those on unclipped culms. If so, enhanced distal floret growth was not due to higher photosynthesis, as these were similar between clipped and unclipped culms, but may have been supported by carbon originating from clipped basal florets (Fig. 2). Determining if this is indeed the case would require additional experimentation, such as comparing differences in distal floret growth above shaded vs unshaded clipped basal florets, as in Hamerlynck and O’Connor (2021).

Most seed mass in rangeland forage grasses is accrued over post-anthesis grain filling following fertilization (McDonald et al. 1996). Our clipping treatments likely removed a significant number of anthers and receptive styles, and thus strongly limited basal flower fertilization, possibly leading to the low specific mass of clipped basal florets (Fig. 4). It is notable that clipped basal florets continued post-anthesis *A*_net_ at levels similar to unclipped counterparts (Fig. 1). Lower specific mass would reduce maintenance respiration (Amthor 1984) being met by *A*_net_ compared to unclipped controls undergoing active seed filling. Clipping therefore may have led to a relaxation of competitive sink demand from the basal portion of the seed head, contributing to the higher distal floret specific mass. Higher proportional distal sink activity may have been met in part by carbon stored over the pre-anthesis assimilated by clipped basal florets, though rangeland grasses typically rely on current photosynthate to support sink activity (Briske and Richards 1995). Rather, it seems probable that photosynthate from clipped basal florets supplemented that assimilated by distal florets over the post-anthesis period. Thus, it seems probable that removing basal floret biomass either induced or modified a source–sink relationship between clipped basal and unaffected distal florets, and that distal sink demand was in part met by photosynthetic activity by basal florets directly affected by tissue loss. In conclusion, this study clearly demonstrates that photosynthetic compensatory behavior is apparent in perennial grass seed heads, and that direct and indirect photosynthetic responses likely facilitate compensatory reproductive responses in these structures. Early research suggested selective pressures for developing reproductive photosynthesis was likely to be strong, as maximizing carbon assimilation in reproductive structures could directly benefit plant fitness (Bazzaz et al. 1979). Our current study suggests compensatory photosynthetic behavior is a critical feature beyond simply having photosynthetically competent floral structures. The presence of compensatory reproductive photosynthesis associated with distinct increases in undamaged floret size and specific mass suggest inconspicuous herbivores may have exerted selective pressure to develop mechanisms to offset total propagule loss in this arid land bunchgrass. Further investigations of the presence and importance of reproductive photosynthetic compensation in native perennial bunchgrasses are needed, given the marked difference in reproductive photosynthetic capacity in these compared to crested wheatgrass (Hamerlynck et al. 2019; Hamerlynck and O’Connor 2022), as well as the greater contributions by flag leaves to reproductive effort in some native grasses (Hamerlynck and O’Connor 2021). Additional studies are also needed to ascertain if the increased floret specific mass in distal florets observed here are driven by actual increases in seed mass and that these improve seedling establishment success. Understanding the role of this dynamic ecophysiological response may be a critical to determining the ability of sagebrush rangeland bunchgrasses to produce seed cohorts capable of germinating and surviving the demographic bottlenecks that typically limit native Great Basin bunchgrasses (James et al. 2011; Hamerlynck and Davies 2019). Moreover, the results of this study present a novel and as yet largely unexplored aspect of the selective pressures driving development of convergent drought- and grazing-tolerance traits that important to the resiliency and persistence of arid and semi-arid rangeland plant communities (Adler et al. 2004; Quiroga et al. 2010), and could be invaluable in selecting and developing native plant materials needed to improve restoration of threatened sagebrush steppe rangeland ecosystems (Davies and Boyd 2021; Hamerlynck and Boyd 2021).

## Supplementary Information

Below is the link to the electronic supplementary material.

Supplementary file1 (TXT 38 KB)

## Data Availability

All data used for this study are available as supplementary material (S1), or upon request to the corresponding author.
